# Insulin resistance is associated with Sfrp5 in obstructive sleep apnea^☆^^☆☆^

**DOI:** 10.1016/j.bjorl.2018.07.002

**Published:** 2018-08-04

**Authors:** Shibo Sun, Huifen Zhai, Mei Zhu, Peili Wen, Xin He, Haoyan Wang

**Affiliations:** a Department of Respiratory Medicine, Beijing Friendship Hospital, Capital Medical University, Beijing, China; b First Department of Respiratory Medicine, First Affiliated Hospital, Kunming Medical University, Kunming, China; c Department of Otolaryngological Medicine, Beijing Friendship Hospital, Capital Medical University, Beijing, China

**Keywords:** Obstructive sleep apnea, Secreted frizzled-related protein 5, Adipokine, Insulin sensitivity, Apneia obstrutiva do sono, Proteína secretada relacionada ao receptor *frizzled* 5, Adipocina, Sensibilidade à insulina

## Abstract

**Introduction:**

Obstructive sleep apnea, a common disease, is usually complicated by insulin resistance and type 2 diabetes mellitus. Adipokine is considered to play an important role in the development of insulin resistance and type 2 diabetes mellitus in obstructive sleep apnea.

**Objective:**

To assess whether secreted frizzled-related protein 5, a new adipokine, is involved in untreated obstructive sleep apnea patients.

**Methods:**

Seventy-six subjects with obstructive sleep apnea and thirty-three control subjects without obstructive sleep apnea were recruited and matched in terms of body mass index and age. The fasting secreted frizzled-related protein 5 plasma concentration was tested using ELISA. In addition, the correlation between secreted frizzled-related protein 5 and the homeostasis model assessment of insulin resistance was obtained. Multiple linear regression analysis models with stepwise selection were performed to determine the independent associations between various factors and secreted frizzled-related protein 5.

**Results:**

Plasma secreted frizzled-related protein 5 levels were significantly lower in the obstructive sleep apnea group than in the control group (obstructive sleep apnea group: 28.44 ± 13.25 ng/L; control group: 34.16 ± 13.51 ng/L; *p* = 0.023). In addition, secreted frizzled-related protein 5 was negatively correlated with homeostasis model assessment of insulin resistance but positively correlated with the mean and lowest oxygen saturation with or without adjusting for age, gender, body mass index, neck circumference, waist circumference and waist-to-hip ratio. The multiple linear regression analysis showed there was an independent negative association between secreted frizzled-related protein 5 and homeostasis model assessment of insulin resistance.

**Conclusion:**

Secreted frizzled-related protein 5 was involved in obstructive sleep apnea and the decrease in secreted frizzled-related protein 5 was directly proportional to the severity of obstructive sleep apnea. There was an independent negative correlation between homeostasis model assessment of insulin resistance and secreted frizzled-related protein 5 in the obstructive sleep apnea group. Secreted frizzled-related protein 5 might be a therapeutic target for insulin resistance in obstructive sleep apnea.

## Resumo

**Introdução:**

A apneia obstrutiva do sono, uma doença comum, é geralmente complicada com resistência à insulina e diabetes melito tipo 2. Acredita-se que a adipocina possa ter um papel importante no desenvolvimento de resistência à insulina e diabetes melito tipo 2 na apneia obstrutiva do sono.

**Objetivo:**

Avaliar se a proteína secretada relacionada ao receptor *frizzled*-5, uma nova adipocina, está envolvida em pacientes com apneia obstrutiva do sono não tratada.

**Método:**

Foram recrutados 76 indivíduos com apneia obstrutiva do sono e 33 indivíduos controle sem apneia obstrutiva do sono e pareados em relação a índice de massa corporal e idade. A concentração plasmática de proteína secretada relacionada ao receptor *frizzled*-5 foi testada em jejum com o teste Elisa. Além disso, obteve-se correlação entre a proteína secretada relacionada ao receptor *frizzled*-5 e o modelo de avaliação da homeostase de resistência à insulina. Modelos de análise de regressão linear múltipla com seleção *stepwise* feitos realizados para determinar as associações independentes entre vários fatores e a proteína secretada relacionada ao receptor *frizzled*-5.

**Resultados:**

Os níveis plasmáticos de proteína secretada relacionada ao receptor *frizzled*-5 foram significativamente menores no grupo com apneia obstrutiva do sono do que no grupo controle (grupo com apneia obstrutiva do sono: 28,44 ± 13,25 ng/L; grupo controle: 34,16 ± 13,51 ng/L; *p* = 0,023). Além disso, a proteína secretada relacionada ao receptor *frizzled*-5 foi correlacionada negativamente com o modelo de avaliação da homeostase de resistência à insulina, mas se correlacionou positivamente com a média e a saturação mínima de oxigênio com ou sem ajuste para idade, gênero, índice de massa corporal, circunferência do pescoço, circunferência da cintura e relação cintura-quadril. A análise de regressão linear múltipla mostrou que houve uma associação negativa independente entre a proteína secretada relacionada ao receptor *frizzled*-5 e o modelo de avaliação da homeostase de resistência à insulina.

**Conclusões:**

A proteína secretada relacionada ao receptor *frizzled*-5 esteve envolvida na apneia obstrutiva do sono e sua diminuição foi diretamente proporcional à gravidade da apneia obstrutiva do sono. Houve uma correlação negativa independente entre o modelo de avaliação da homeostase de resistência à insulina e a proteína secretada relacionada ao receptor *frizzled*-5 no grupo da apneia obstrutiva do sono. A proteína secretada relacionada ao receptor *frizzled*-5 pode ser um alvo terapêutico para a resistência à insulina na apneia obstrutiva do sono.

## Introduction

Obstructive sleep apnea (OSA) is a common breathing disorder. Its prevalence is high with a mean of 22% in men and 17% in women.^1^ OSA is characterized by the repetitive cessation of breathing due to airway obstruction during sleep, leading to hypoxemia and sleep disruptions.^2^ Studies showed OSA was associated with metabolic disorders, such as insulin resistance (IR), type 2 diabetes (T2DM).^3^, ^4^, ^5^ The exact mechanisms underlying this association remain unclear. However, adipose tissue is considered to play an important role in the development of IR and T2DM in OSA.^6^

Adipose tissue is not only an energy storage tissue but also an endocrine organ that secretes various adipokines.^7^ Increasing evidence shows that adipokine abnormalities are involved in OSA.^8^, ^9^, ^10^ As an adipokine, secreted frizzled-related protein 5 (Sfrp5) is highly expressed in white adipose tissue.^11^, ^12^, ^13^, ^14^ Sfrp5 is associated with IR, T2DM and coronary artery diseases, and even represents a potential therapeutic target for abnormal glucose homeostasis.^11^, ^15^, ^16^, ^17^, ^18^ In addition, Sfrp5 expression is down-regulated under conditions of oxidative stress,^19^ which are frequently present in OSA during sleeping because of intermittent hypoxia.^20^ Thus, we speculated the changes in Sfrp5 might be involved in OSA and be associated with IR in OSA. Assessing this association might contribute to identifying the mechanisms of IR in OSA and is thus crucial for the preventing from risks of IR and T2DM in OSA.

## Methods

### Subjects

All subjects were recruited from the Sleep Laboratory of the Department of Respiratory Medicine, the Department of Otolaryngological Medicine or the Health Examination Center of Beijing Friendship Hospital from 2016.03 to 2017.04. The inclusion criterion was being age from 18 to 70 years old. The exclusion criteria were having metabolic, allergic, cardiovascular, cerebrovascular, renal, or liver diseases, psychiatric disorders, having infectious diseases of the lungs, sleep disorders other than OSA, being pregnant, and having been previously treated for OSA. The study was approved by the Ethics Committee of Beijing Friendship Hospital and informed consent was obtained from all participants. The ethics code number: 2016-P2-003-02.

All subjects underwent a thorough physical examination before undergoing polysomnography (PSG). neck circumference (NC), waist circumference (WC), waist-to-hip ratio (WHR), and body mass index [BMI, weight (height)^2^] were measured. In addition, subjects were classified as obese, non-obese.^21^ The Epworth Sleepiness Score (ESS) was collected to assess the level of daytime sleepiness.

### PSG

All subjects underwent overnight PSG using a sleep data-acquisition system (Homlogic N7000, Australia). Briefly, the recorded parameters were electroencephalograms from leads applied at C4-M1, C3-M2, O2-M1, O1-M2, F4-M1, and F3-M2, respiratory airflow detected by a flow pressure receptor, respiratory movements detected by thoracic and abdominal bands, oxygen saturation detected by a pulse oximeter, electrooculograms, as well as chin and anterior tibial electromyograms. Apnea was defined as an amplitude reduction >90% of nasal airflow for at least 10s. Hypopnea was defined as an airflow amplitude reduction >30% associated with oxyhemoglobin desaturation of 3% or more or associated with arousal. All PSG records were analyzed by two specialists. Normal subjects were defined as those having an Apnea/Hypopnea Index (AHI) < 5, and subjects with OSA were defined as those having an AHI ≥ 5.

### Blood assay

Venous blood samples were obtained from all subjects in the morning after PSG and after having fasting overnight. The plasma was extracted by centrifugation at 1000 g/min for 20 min and stored at −80 °C until measurement. Plasma Sfrp5 was assessed using an ELISA kit (ShinnyBIO, Shanghai, China). All other blood tests (triglyceride [TG], total cholesterol [TC], low density lipoprotein [LDL], high density lipoprotein [HDL], high-sensitivity C-reactive protein [HS-CRP], fasting blood glucose, Fasting insulin) were completed by the Beijing Friendship Hospital. Insulin sensitivities were assessed using the homeostasis model assessment of insulin resistance (HOMA-IR) = (fasting glucose [mmoL/L] * fasting insulin [lU/mL])/22.5.^22^

### Statistical analysis

Data are presented as the mean ± Standard Derivation for continuous variables and the number for categorical variables. One-sample Kolmogorov–Smirnov tests were performed to determine whether the independent variables were normally distributed. Comparisons of independent groups were performed with the independent-samples Student's *t*-test or the Mann–Whitney *U*-test based on the normality distribution. Categorical variables were compared with the Chi-square test. Linear correlations were analyzed by Spearson's correlation coefficient. A multiple linear regression analysis with stepwise selection was used to determine associations between various factors and Sfrp5. *p*-value <0.05 was considered to indicate significance. The statistical analyses of the data were conducted using SPSS 17.0.

## Results

109 subjects were divided into two groups: the OSA group (n = 76; age: 46.13 ± 11.61 years; male/female: 56/20) and the control group (n = 33; age: 42.21 ± 11.22 years; male/female: 20/13). According to AHI, the OSA groups were divided into three subgroup: mild OSA (5 ≤ AHI < 15), moderate OSA (15 ≤ AHI < 30), severe OSA (AHI ≥ 30). All baseline characteristics and blood testing results of the subjects are presented in Table 1, Table 2.

**Table 1:** Baseline demographics, sleep profiles and blood measurements of the control and OSA groups.

	Control group (*n* = 33)	OSA group (*n* = 76)	*p*-value
Age (years)	42.21 ± 11.22	46.13 ± 11.61	0.111
Gender (male/female)^a^	20/13	56/20	0.182
Body mass index (kg/m^2^)	27.50 ± 5.15	28.90 ± 4.65	0.184
Neck circumference (cm)	37.19 ± 4.35	40.71 ± 4.27	<0.001^b^
Waist circumference (cm)	91.67 ± 9.25	95.59 ± 8.66	0.036^b^
Waist-to-hip ratio	0.89 ± 0.06	0.93 ± 0.05	0.001^b^
Apnea/hypopnea index (events/h)	2.56 ± 1.25	44.10 ± 27.76	<0.001^b^
Lowest oxygen saturation (%)	90.01 ± 2.78	78.49 ± 9.70	<0.001^b^
Mean oxygen saturation (%)	93.45 ± 1.29	89.73 ± 4.28	<0.001^b^
Sfrp5 (ng/L)	34.16 ± 13.51	28.44 ± 13.25	0.023^b^
Triglycerides (mmoL/L)	1.65 ± 0.51	2.08 ± 1.87	0.070
Total cholesterol (mmoL/L)	4.64 ± 1.50	4.83 ± 2.00	0.629
Low-density lipoprotein (mmoL/L)	2.68 ± 0.94	2.62 ± 0.71	0.707
High-density lipoprotein (mmoL/L)	1.17 ± 0.34	1.14 ± 0.62	0.762
HS-CRP (mg/L)	2.27 ± 3.07	3.34 ± 3.91	0.213
Fasting blood glucose (mmoL/L)	4.65 ± 0.72	4.93 ± 0.67	0.052
Fasting insulin (IU/mL)	17.39 ± 8.53	19.93 ± 6.87	0.102
HOMA-IR	3.55 ± 1.72	4.32 ± 1.49	0.020^b^
Epworth sleepiness score	7.70 ± 4.28	12.34 ± 3.89	<0.001^b^

Values are indicated as the mean ± standard deviation.

Sfrp5, Secreted frizzled-related protein 5; HS-CRP, High-sensitivity C-reactive protein; HOMA-IR, Homeostasis Model Assessment of Insulin Resistance.

a Value is presented in numbers.

b *p* < 0.05, statistically significant difference.

**Table 2:** Baseline demographics, sleep profiles and blood measurements of the control and OSA subgroups.

	Control (*n* = 33)	Mild (*n* = 12)	Moderate (*n* = 20)	Severe (*n* = 44)
Age (years)	42.21 ± 11.22	43.83 ± 14.41	50.95 ± 8.37^a^	44.56 ± 11.67
BMI (kg/m^2^)	27.50 ± 5.15	29.44 ± 5.23	27.86 ± 3.59	29.23 ± 4.93
NC (cm)	37.19 ± 4.35	39.29 ± 4.88	39.15 ± 3.58	41.80 ± 4.13^a^
WC (cm)	91.67 ± 9.25	94.33 ± 9.23	92.47 ± 7.91	97.35 ± 8.56^a^
WHR	0.89 ± 0.06	0.90 ± 0.05	0.91 ± 0.04	0.94 ± 0.05^a^
AHI (events/h)	2.56 ± 1.25	10.36 ± 3.51^a^	21.87 ± 5.12^a^	63.41 ± 24.50^a^
Lowest oxygen saturation (%)	90.01 ± 2.78	87.17 ± 3.51^a^	83.90 ± 5.41^a^	73.66 ± 9.46^a^
Mean oxygen saturation (%)	93.45 ± 1.29	92.85 ± 1.74	91.03 ± 2.53^a^	88.28 ± 4.77^a^
Sfrp5 (ng/L)	34.16 ± 13.51	30.60 ± 15.03	29.99 ± 14.82	27.13 ± 12.13^a^
Triglycerides (mmoL/L)	1.65 ± 0.51	1.57 ± 0.89	2.00 ± 0.99	2.25 ± 2.32
Total cholesterol (mmoL/L)	4.64 ± 1.50	4.59 ± 0.88	4.75 ± 0.76	4.94 ± 2.54
Low-density lipoprotein (mmoL/L)	2.68 ± 0.94	2.71 ± 0.79	2.65 ± 0.68	2.58 ± 0.71
High-density lipoprotein (mmoL/L)	1.17 ± 0.34	1.14 ± 0.48	1.14 ± 0.32	1.14 ± 0.76
HS-CRP (mg/L)	2.27 ± 3.07	2.26 ± 2.85	2.47 ± 2.82	4.01 ± 4.47^a^
Fasting blood glucose (mmoL/L)	4.65 ± 0.72	4.99 ± 0.86	4.83 ± 0.60	4.95 ± 0.65
Fasting insulin (IU/mL)	17.39 ± 8.53	16.48 ± 6.10	17.95 ± 5.00	21.78 ± 7.29^a^
HOMA-IR	3.55 ± 1.72	3.55 ± 1.72	3.78 ± 0.95	4.74 ± 1.54^a^
ESS	7.70 ± 4.28	9.83 ± 3.10	10.80 ± 2.71^a^	13.73 ± 3.99^a^

Values are indicated as the mean ± standard deviation.

BMI, Body mass index; NC, Neck circumference; WC, Waist circumference; WHR, Waist-to-hip ratio; AHI, Apnea/hypopnea index; Sfrp5, Secreted frizzled-related protein 5; HS-CRP, High-sensitivity C-reactive protein; HOMA-IR, Homeostasis Model Assessment of Insulin Resistance; ESS, Epworth sleepiness score.

a *p* < 0.05 vs. the control.

There was significant difference in Sfrp5 levels between the control and OSA groups (OSA group: 28.44 ± 13.25 ng/L; control group: 34.16 ± 13.51 ng/L; *p* = 0.023). The concentrations of Sfrp5 were significantly lower in severe OSA group than control group (Table 2).

**Figure 1:**
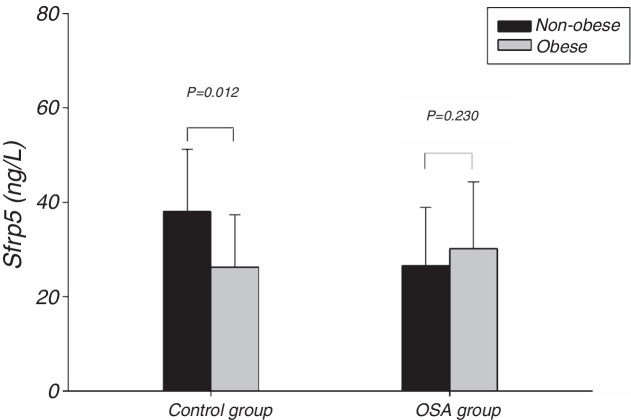
Comparison of Sfrp5 levels between non-obese and obese in the control and OSA groups. OSA, obstructive sleep apnea; Sfrp5, secreted frizzled-related protein 5.

**Figure 2:**
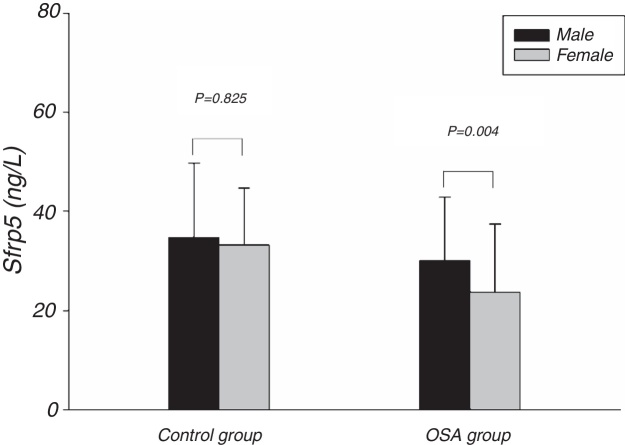
Comparison of Sfrp5 levels between different genders in the control and OSA groups. OSA, obstructive sleep apnea; Sfrp5, secreted frizzled-related protein 5.

Fig. 1 showed the Sfrp5 level in control group was significantly lower in obese subjects than in non-obese subjects. In addition, there was no difference in Sfrp5 levels between males and females in control group (Fig. 2).

The correlation analysis showed that the Sfrp5 level was negatively correlated with BMI, ESS HS-CRP, HOMA-IR but positively correlated with the lowest and mean oxygen saturation levels. Adjusting for age, gender, BMI, NC, WC, and WHR, Sfrp5 was still negatively correlated with HOMA-IR and ESS (Table 3). The multiple linear regression analysis showed there was an independent negative correction between HOMA-IR and Sfrp5 in the OSA group (Table 4).

**Table 3:** Spearman's correlations between Sfrp5 and the other factors.

	*r*	*p*-value	*r*^a^	*p*-value	*r*^b^	*p*-value
Age	−0.094	0.329				
Body mass index	−0.284	0.003^a^				
Neck circumference	−0.211	0.028^a^	−0.087	0.375		
Waist circumference	−0.147	0.127	0.055	0.576		
Waist-to-hip ratio	−0.063	0.513		0.401		
Apnea/hypopnea index	−0.170	0.076	−0.099	0.312	−0.125	0.205
Lowest oxygen saturation	0.236	0.013^a^	0.193	0.048^a^	0.211	0.032^a^
Mean oxygen saturation	0.321	0.001^a^	0.223	0.022^a^	0.251	0.011^a^
Triglycerides	−0.058	0.551	−0.107	0.274	−0.130	0.190
Total cholesterol	−0.000	0.993	−0.077	0.432	−0.055	0.583
Low-density lipoprotein	0.048	0.624	0.160	0.102	0.184	0.063
High-density lipoprotein	−0.012	0.901	−0.175	0.073	−0.155	0.119
HS-CRP	−0.219	0.022^a^	−0.098	0.319	−0.100	0.318
Fasting blood glucose	−0.117	0.227	−0.123	0.208	−0.110	0.268
Fasting insulin (mU/L)	−0.205	0.032^a^	−0.166	0.089	−0.210	0.033^a^
HOMA-IR	−0.243	0.011^a^	−0.215	0.027^a^	−0.253	0.010^a^
Epworth sleepiness score	−0.237	0.013^a^	−0.265	0.006^a^	−0.272	0.005^a^

a Adjusting for age, BMI, gender.

b Adjusting for age, gender, Body mass index; Neck circumference; Waist circumference; Waist-to-hip ratio. Sfrp5, Secreted frizzled-related protein 5; HS-CRP, High-sensitivity C-reactive protein; HOMA-IR, Homeostasis Model Assessment of Insulin Resistance. *p < 0.05, Statistically significant difference.

**Table 4:** Stepwise multiple regression models of Sfrp5 levels in the control and OSA groups.

	Control group Adjusted (*R*^2^ = 64.9%)			OSA group Adjusted (*R*^2^ = 5.4%)		
	*B* (SE)	*β*	*p*-value	*B* (SE)	*β*	*p*-value
Constant	69.872 (17.791)		0.000	38.338 (4.558)		0.000
BMI	−0.963 (0.293)	−0.367	0.003			
HDL	−13.814 (4.477)	−0.350	0.005			
AHI	7.686 (1.299)	0.709	0.000			
HOMA-IR	−3.586 (0.960)	−0.456	0.001	−2.389 (0.997)	−0.258	0.024

BMI, Body mass index; HDL, High-density lipoprotein; AHI, Apnea/hypopnea index; HOMA-IR, Homeostasis Model Assessment of Insulin Resistance.

Independent variables considered: age, gender, BMI, epworth sleepiness score (ESS), AHI, High-sensitivity C-reactive protein (HS-CRP), HOMA-IR, triglycerides (TG), total cholesterol (TC), low-density lipoprotein (LDL), high-density lipoprotein (HDL).

## Discussion

The present study showed that the Sfrp5 concentrations were significantly decreased in OSA. In addition, the Sfrp5 was positively correlated with the lowest and mean oxygen saturations with or without adjustments for age, gender, BMI, NC, WC and WHR. These results suggest that Sfrp5 might be involved in the pathophysiological process of OSA. Previous studies have reported that hypoxia could induce necrosis in 3T3-L1 adipocytes^23^ and increase adipocytes death in the adipose tissue of obese subjects.^24^ Most importantly, the expression of Sfrp5 was down-regulated under the conditions of oxidative stress.^19^ Undoubtedly, hypoxia followed by oxidative stress occurs frequently during sleep^25^ in OSA. It seems that the reduction in circulating Sfrp5 may be a result of the adipocyte death and oxidative stress caused by hypoxia in OSA. However, Zhang DM^26^ suggested that the Sfrp5 levels of OSA patients did not differ from that of non-OSA individuals. In the study of Zhang DM, the subjects were severe OSA individuals whose AHI were lower than that of severe OSA in present study. Importantly, Zhang DM's study, unlike the present study, did not include females. In addition, the present study showed the Sfrp5 concentrations of females were lower than that of males in OSA group (Fig. 2). To some extent, the discrepancies among these studies might lie in the different clinical characteristics of the studies.

One study showed that Sfrp5 expression was decreased in the adipose tissue of obese mice.^11^ Studies^14^, ^15^, ^27^ suggested that there was a significantly lower circulating Sfrp5 concentration under conditions of obesity than under conditions of normal weight. In the present study, plasma Sfrp5 levels were significantly lower in obese subjects than in normal weight subjects, and Sfrp5 was negatively correlated with BMI. Furthermore, the multiple linear regression analysis showed that the BMI independently predicted the Sfrp5 level in control group. We speculated that the Sfrp5 expression might be reduced in obese humans, although this hypothesis needs to be verified in future studies.

As an anti-inflammatory adipokine, Sfrp5 inhibits the accumulation of activated macrophages in adipose tissue via noncanonical regulation of the JNK signaling pathway to ameliorate glucose intolerance in mouse models of obesity and T2DM.^11^ Studies have reported that Sfrp5 is inversely correlated with HOMA-IR in obese subjects with normal glucose tolerance or T2DM.^12^, ^15^, ^18^ We also found a significantly negative correlation even when the confounding factors of age, gender, BMI, NC and WHR were eliminated. Most importantly, the multiple linear regression analysis showed the negative association independently between Sfrp5 and IR in the OSA group. We speculated the reason for the negative correlation was that the decrease in Sfrp5 might attenuate its anti-inflammatory effects in adipose tissue in OSA.

Some studies suggested that adipokine levels might be correlated with gender.^28^, ^29^ However, we found no differences in the plasma Sfrp5 level between women and men in control group. In agreement with our finding, previous studies have also failed to find an association between the serum Sfrp5 levels and the sex of the subjects.^15^, ^16^, ^17^

The main limitation of present study was that the study was followed a case-control design without randomization, blinding or a placebo control.

## Conclusions

In conclusion, Sfrp5 was involved in OSA and the decrease in Sfrp5 was directly proportional to the severity of OSA. There was an independent negative correlation between HOMA-IR and Sfrp5 in the OSA group. Sfrp5 might be a therapeutic target for IR in OSA.

## Funding

The study was funded by Natural Science Foundation of Beijing Municipality (CN) (No. 7142046), Teaching and Reform Program of Kunming Medical University (No. 2016-JY-Y-43), and Yunnan Provincial Department of Education (No. 2017zzx201).

## Conflicts of interest

The authors declare no conflicts of interest.
